# Implementing a Group Psychoeducational Program for Emotional Well-Being in Primary Care Teams: A Qualitative Study in Catalonia

**DOI:** 10.3390/healthcare14030402

**Published:** 2026-02-05

**Authors:** Enric Aragonès, Sara Rodoreda, Meritxell Guitart, Eva Garcia, Anna Berenguera, Francisco Martín-Luján, Concepció Rambla, Guillem Aragonès, Antoni Calvo, Ariadna Mas, Dolors Rodríguez, Josep Basora

**Affiliations:** 1 Primary Care Area Camp de Tarragona, Institut Català de la Salut, 43005 Tarragona, Spain; fmartin.tgn.ics@gencat.cat (F.M.-L.); crambla.tgn.ics@gencat.cat (C.R.); 2 Institut Universitari d’Investigació en Atenció Primària Jordi Gol (IDIAPJGol), 08008 Barcelona, Spain; aberenguera@idiapjgol.org (A.B.); garagones@idiapjgol.info (G.A.); lrodrigu.ar@gmail.com (D.R.); jbasora@idiapjgol.org (J.B.); 3 Primary Care Division, Institut Català de la Salut, 08008 Barcelona, Spain; srodoreda.mn.ics@gencat.cat; 4 Primary Care Emotional and Community Well-Being Program, Department of Health, Government of Catalonia, 08028 Barcelona, Spain; mguitartp.cc.ics@gencat.cat; 5 Primary Care Center Sant Vicenç de Castellet, Institut Català de la Salut, 08295 Barcelona, Spain; 6 Occupational Risk Prevention Unit, Institut Català de la Salut, 08008 Barcelona, Spain; egarciacots@gencat.cat; 7 Department of Medicine and Surgery, School of Medicine and Health Sciences, Universitat Rovira i Virgili University, 43201 Reus, Spain; 8 Transversal Lifestyle and Disease Prevention Program, Intitut d’Investigació Sanitària Pere Virgili (IISPV), 43204 Tarragona, Spain; 9 Galatea Foundation, 08017 Barcelona, Spain; tcalvo@comb.cat; 10 General Directorate of Health Planning and Research, Department of Health, Government of Catalonia, 08028 Barcelona, Spain; amasc@gencat.cat

**Keywords:** primary health care, healthcare workers, qualitative research, burnout, psychosocial support, psychological resilience, occupational stress, organizational support

## Abstract

**Highlights:**

**What are the main findings?**

- A psychoeducational emotional well-being program was well received by primary care professionals, providing a space for emotional expression and self-care.
- Community psychologists played a key role in facilitating the intervention, while organizational barriers limited participation and continuity.

**What are the implications of the main findings?**

- Sustaining emotional well-being initiatives in primary care requires organizational commitment, adequate resources, and integration into routine practice.
- Combining individual-level support with systemic measures addressing workload, staffing, and organizational culture is essential.

**Abstract:**

**Background/Objectives**: Healthcare workers have faced increasing emotional strain driven by organizational constraints, rising workload, and accumulated post-pandemic pressure. To support emotional well-being in primary care professionals, the Catalan Health Institute implemented a large-scale psychoeducational group program in its primary care centers. This study explored its feasibility, acceptability, and the factors shaping real-world implementation from the perspectives of participating professionals and community psychologists who taught it. **Methods**: A qualitative study was conducted involving five online focus groups held with community psychologists (two groups) and primary care professionals who participated in the program (three groups), selected through purposive sampling. Additional qualitative material was obtained from implementation-related field notes. Session transcripts were analyzed using inductive thematic analysis. The study is registered at ClinicalTrials.gov (NCT05720429). **Results**: Participants described a context of sustained emotional strain that increased motivation to engage with the program. The sessions were perceived as a valuable protected space for emotional expression, interpersonal connection, and learning self-care strategies. Community psychologists were regarded as key facilitators due to their embedded role and contextual knowledge. However, inconsistent managerial engagement, lack of protected time, competing workloads, and inadequate physical spaces were barriers to successful implementation. Participants proposed strengthening institutional support and offering follow-up sessions to consolidate benefits. **Conclusions**: The program was positively valued and was perceived to provide individual and team-level benefits. Its sustainability requires stronger organizational commitment and integration into routine practice. Findings underscore the need to complement individual-focused interventions with systemic actions addressing workload, staffing, and organizational culture.

## 1. Introduction

The mental health of healthcare professionals has become a central concern in global public health agendas [1]. Primary care teams, in particular, have been exposed to a progressive intensification of workload, increasing clinical complexity, bureaucratic demands, and pressure to maintain accessibility and quality of care [2,3]. This scenario has led to growing emotional distress, significantly exacerbated by the COVID-19 pandemic [4,5]. In Catalonia, a survey conducted in mid-2021 among family physicians revealed that nearly two-thirds of respondents showed high levels of emotional exhaustion [6]. New surveys conducted in 2022 and 2023 showed a slight decrease in burnout prevalence over time, but over half of participants consistently reported elevated levels of emotional exhaustion, indicating that burnout remains a significant concern in primary care [7]. Similar findings have been reported internationally, confirming that workload, uncertainty, and lack of institutional recognition are key contributors to deteriorating emotional well-being among healthcare workers [8,9].

In this context, the notion of “caring for the carers” has gained prominence, emphasizing the need to protect the emotional health of professionals as a prerequisite for delivering quality care [10,11]. However, institutional responses have varied widely, ranging from individual-focused approaches, such as promoting self-care, emotional regulation, and mindfulness, to broader proposals that call for structural changes in working conditions, organizational culture, and leadership models [11].

Group-based psychoeducational interventions have emerged as a promising strategy to promote emotional health in healthcare settings [12,13]. Evidence suggests that such interventions can enhance team cohesion, reduce perceived stress, and provide structured spaces for emotional release. Nevertheless, their implementation in real-world clinical environments requires careful alignment with work dynamics, institutional support, and organizational culture [14]. Ensuring continuity over time and integrating these interventions into routine practice depends on multiple factors that often go beyond individual motivation. The tension between fostering individual resilience and addressing systemic determinants is a critical issue that must be considered when evaluating the impact and sustainability of emotional well-being interventions [15].

In response to these challenges, the Catalan Health Institute (ICS) has developed and deployed a group psychoeducational intervention facilitated by community psychologists, referred to as RBEC (Community Emotional Well-Being Lead, in Catalan: Referent de Benestar Emocional Comunitari), which is offered to professionals in all its primary care centers in Catalonia. The program aims to promote emotional well-being through structured sessions adapted to the specific needs of each team and integrated into regular work schedules.

This study presents an exploratory qualitative evaluation of the program’s real-world implementation. We aimed to understand participants’ experiences focusing on the feasibility, acceptability, and contextual appropriateness of the intervention, contributing to the broader debate on how to effectively address emotional distress among healthcare professionals. Specifically, we addressed two research questions: (1) How did primary care professionals and RBEC psychologists experience the program in routine practice, and what aspects were perceived as most useful or challenging? (2) What organizational and contextual conditions were perceived to enable participation and support the program’s continuity over time?

## 2. Materials and Methods

### 2.1. Study Design

This study evaluates the real-world implementation of a group psychoeducational intervention aimed at promoting emotional well-being and managing psychological distress among primary care professionals. The research was part of a broader mixed-methods project, described in detail in a previously published protocol [16], but the present article reports exclusively on the qualitative component. The qualitative study was conducted from a phenomenological interpretive perspective, recognizing that participants’ accounts reflect both their lived experiences and the organizational contexts in which the program was delivered. This orientation supported an inductive analytic approach aimed at identifying patterns, underlying mechanisms, and contextual factors influencing how the intervention was implemented and experienced across diverse primary care settings [17]. Consistent with its inductive design, this qualitative component was not guided by an a priori implementation framework.

The study protocol was approved by the Clinical Research Ethics Committee of the Jordi Gol Institute for Primary Care Research (Barcelona, 27 May 2022; code 22/086-PCV). All participants provided informed consent prior to data collection. The study is registered at ClinicalTrials.gov (NCT05720429) and follows the Consolidated Criteria for Reporting Qualitative Research (COREQ) [18]. A detailed COREQ checklist is provided in the Supplementary Materials.

### 2.2. Setting and Participants

The psychoeducational program was implemented between September 2022 and February 2024 in 81 primary care centers of the Catalan Health Institute, as part of the wider Emotional Well-being and Community Health Program [19], an initiative of the Catalan Department of Health. The intervention was open to all primary care professionals, including family doctors, nurses, pediatricians, dentists, physiotherapists, nutritionists, social workers and administrative staff [20]. During the study period, a total of 1419 professionals participated in the psychoeducational groups.

For the qualitative sub-study, purposive sampling was used to recruit two key stakeholder groups: community psychologists (RBECs) who delivered the intervention and primary care professionals who had participated in a full or partial cycle of sessions. Sampling aimed to ensure variation in professional background, gender, age, territorial context, and degree of adherence to the intervention. Participants were identified through program coordinators and local teams. Because participation required consent and scheduling availability, recruitment relied on volunteers who confirmed their willingness to take part, which may have introduced self-selection. To mitigate this, we sought maximum variation in professional role, gender, age, territorial context, and degree of adherence (including professionals who completed a full or partial cycle of sessions). This heterogeneity enabled a rich understanding of experiences, expectations, and contextual factors shaping implementation across diverse primary care settings. In total, 38 participants (18 RBECs and 20 participating professionals) contributed to the focus groups. Participant characteristics are summarized in Table 1.

### 2.3. Intervention

The intervention is a structured group psychoeducational program designed by a collaborative working group of expert psychologists. It integrates evidence-based psychological and psychoeducational strategies aimed at improving emotional well-being, preventing mental health problems, and fostering peer support within primary care teams. The program consists of 11 in-person weekly or biweekly sessions lasting 45–60 min, delivered during working hours by community psychologists affiliated with primary care centers. Each session follows a structured format including a brief theoretical introduction, experiential activities, and a guided meditation. Although it was based on a standardized protocol, adaptations to local needs were contemplated. The content of the sessions is summarized in Table 2, and a more detailed description has been published.

### 2.4. Data Collection

Data collection involved five online focus groups conducted between June and December 2023. Two groups involved RBEC community psychologists (FG1, n = 10; FG2, n = 8). Three groups involved primary care professionals who participated in the program (FG3, n = 10; FG4, n = 6; FG5, n = 4) (Table 1). Given the geographical dispersion of centers, all sessions were conducted via Microsoft Teams and lasted between 58 and 91 min.

Each focus group followed a semi-structured discussion guide (Table 3) exploring perceptions of the program’s usefulness, feasibility, and acceptability; experiences with group dynamics; contextual barriers and facilitators; and suggestions for improvement. The guide allowed moderators to adapt prompts according to participants’ contributions.

All focus groups were recorded with consent, transcribed verbatim, and pseudonymized. Transcripts were not returned to participants for comment and/or correction. They were stored on secure IDIAPJGol servers in compliance with European data protection regulations.

Sessions were moderated by an experienced qualitative researcher (D.R.) and observed by a second qualitative researcher (G.A.), both certified experts in qualitative methods. No external individuals were present; specifically, no other researchers, study promoters, or institutional leadership members attended the sessions. D.R. is a female sociologist and freelance qualitative researcher, with extensive training and experience in conducting qualitative studies in health and social care contexts. G.A. is a male graduate in Philosophy, Politics, and Economics, working as a research support technician at IDIAP, with training in qualitative data collection and analysis. No prior relationship had been established between D.R. and the participants. However, participants knew G.A. because of his involvement in the project’s development. At the beginning of each session, participants were informed that the researchers were external to the organizational structures of the participating centers and that the study aimed to explore professionals’ experiences and perceptions regarding the program. The researchers declared no personal or professional conflicts of interest with the research topic. They also reflexively acknowledged their background in primary care and health research and, in the case of G.A., his prior familiarity with some participants through his role in the project development team as potential sources of interpretive influence.

In addition to the focus groups, qualitative material was gathered through field notes collected during the entire implementation process. These notes included observations, contextual descriptions, informal exchanges with stakeholders, logistical considerations, and methodological reflections. Field notes were used to enrich contextual understanding, help interpret tensions or contradictions emerging during the focus groups, and, when relevant, were coded and analyzed alongside the transcripts.

### 2.5. Data Analysis

A thematic analysis [17] was conducted on all qualitative materials, including focus group transcripts and implementation-related field notes. An inductive approach was applied to ensure that the analysis remained grounded in participants’ lived experiences. The analytic process included: initial immersive reading of the material; identification of salient topics; segmentation into meaningful units; coding through both initial descriptive and emergent codes; and clustering of codes into broader analytical categories to identify patterns, convergences, and divergences. Coding was conducted independently by two researchers (D.R. and G.A.), who subsequently compared and refined codes to ensure consistency. We did not conduct profession-specific comparisons, as the study was exploratory and the professional sub-sample was not designed to support meaningful between-profession analyses. Although coding and theme development were inductive, we subsequently mapped the emergent domains to implementation-relevant outcome areas (feasibility, acceptability, adoption, sustainability) to support clearer reporting. This mapping was applied at the reporting stage and did not guide data collection or coding.

Field notes played a complementary role by providing contextual nuances, informal insights, and implementation-process information that enriched and triangulated the material obtained in the focus groups.

Triangulation was applied at two levels to enhance the credibility and trustworthiness of the findings: data triangulation, by integrating information from multiple sources (psychologists, healthcare professionals, and field notes), and analyst triangulation, through collaborative interpretation by several members of the research team, with discrepancies discussed until consensus was reached, resulting in a single coding tree. Saturation was deemed to have been reached in the fifth focus group, when no substantially new themes or conceptual insights emerged.

No specialized software was used for data management, and participants were not asked to review the findings.

## 3. Results

The analysis revealed a set of interconnected themes describing how the intervention was integrated into everyday practice, how contextual conditions shaped participation, and how both community psychologists and primary care professionals perceived its benefits and limitations. Together, these findings offer a nuanced understanding of the factors that supported or hindered implementation and of the ways in which the program influenced emotional well-being and team dynamics.

The thematic analysis identified six interrelated domains that characterize how the intervention was experienced and implemented across primary care centers. These domains are summarized in Table 4 and examined in detail below. For reporting purposes, these domains are also linked to core implementation outcomes (feasibility, acceptability, adoption, and sustainability) in Table 4.

### 3.1. Pre-Existing Conditions and Emotional Context

Participants described a sustained climate of high workload, accumulated emotional strain, and post-pandemic fatigue, which served both as a vulnerability factor and a motivation to participate. A nurse stated: *“COVID was tough enough already, because it also meant dealing with everything that suddenly hit all of us at once.”* (PC28, FG4). Many perceived the sessions as arriving at a moment of “emotional saturation” in which reflection and decompression were urgently needed. As one RBEC noted, *“Understanding our role was already difficult, and the information didn’t always help”* (RBEC7, FG1).

Pre-existing team cohesion shaped early engagement with the sessions. One professional explained, *“This time helped with group cohesion”* (PC33, FG3). By contrast, a tense atmosphere or poor team dynamics could make successful implementation of the program more difficult.

Beyond these initial conditions, participants highlighted several organizational and interpersonal factors that shaped their engagement with the sessions.

### 3.2. Facilitating Factors and Institutional Support

The RBEC role was consistently described as central to the program’s success. Participants valued their familiarity with the centers and ability to foster trust, as captured in this quote: *“The psychologist was very dynamic and approachable; that kept us motivated”* (PC25, FG5). However, some RBECs initially expressed reservations about working with their own teams, citing concerns related to role boundaries and professional distance. Despite these initial concerns, in most cases the RBEC’s proximity and prior knowledge of the team were ultimately perceived as key facilitators, enabling greater trust and engagement. More broadly, RBEC psychologists were perceived to offer more than simple “internal facilitation”: their embedded role was described as fostering trust and psychological safety through familiarity with the center and its day-to-day pressures. Continuity was also described as enabling tailoring to team dynamics and informal follow-up.

Institutional support, especially explicit managerial endorsement, was also decisive. As one participant noted, *“…the center’s management made it easy for us, during working hours, to avoid home visits or anything like that”* (PC24, FG5). Protected time facilitated attendance when managers actively prioritized the sessions.

These organizational facilitators intersected with the way participants experienced the content and structure of the sessions themselves. As managerial staff were not interviewed, these leadership-related facilitators and barriers reflect participants’ accounts and perceptions rather than managers’ own perspectives.

### 3.3. Perceptions of Content and Group Dynamics

Participants clearly preferred experiential activities, which were perceived as immediately applicable. One RBEC explained, *“The presentations were very theoretical and long; I reduced the theory and focused on practice”* (RBEC4, FG1). Consistent with this, a professional commented, *“The most useful session was the last one, without slides”* (PC32, FG3).

The multiprofessional format promoted mutual understanding. As one RBEC described, *“…this relationship of mutual listening between each other, of mutual help, and of working together as a group”* (RBEC18, FG2).

These experiential preferences and group dynamics strongly shaped how participants perceived the impact of the program.

### 3.4. Perceived Benefits and Impact

Professionals reported that the sessions provided a safe space for emotional ventilation and temporary relief from a demanding work environment. They also described reactivating self-care strategies that had been neglected. As one RBEC summarized, *“Professionals realized that to care for others, they need to care for themselves”* (RBEC18, FG2).

Team-level benefits included improved communication and renewed cohesion, expressed in quotes such as *“We realized we needed a bit more cohesion in our team”* (PC33, FG3).

RBEC psychologists also experienced increased visibility, particularly relevant given that their role is newly implemented in primary care teams and still relatively unknown and weakly integrated. As one RBEC noted: *“Indirectly, I gained visibility and presence in the primary care team”* (RBEC1, FG1).

However, these positive experiences coexisted with several structural and logistical challenges.

### 3.5. Challenges and Barriers

Participants identified several factors that limited the consistency and reach of the program across centers. Lack of protected time remained the most recurrent difficulty, often leading to irregular attendance. As one RBEC stated, *“Schedules weren’t closed, so even if they had the time, they couldn’t attend”* (RBEC5, FG1).

Physical space constraints posed additional challenges. *“The space we used at the center, in the physiotherapy room, lacked privacy due to a corridor and people passing by”* (RBEC13, FG2).

Uneven institutional support and confusion with overlapping initiatives in some territories further complicated implementation. Some participants raised concerns that the program could not compensate for broader structural problems. As one RBEC conveyed, several participants highlighted that, “*What we need more than this program is additional staff*” (RBEC15, FG2). Another recalled explicit resistance from some professionals: *“… they said things like, ‘I would rather you take care of the population than of us’”* (RBEC14, FG2). These accounts highlight a perceived mismatch between the program’s individual-level support and the structural drivers of distress. For some participants, the intervention risked being interpreted as symbolic mitigation if not accompanied by organizational measures addressing workload and staffing.

Alongside these difficulties, participants proposed several ways to strengthen and sustain the intervention over time.

### 3.6. Improvement Proposals and Future Perspectives

Participants expressed a clear interest in maintaining and strengthening the program. Many recommended periodic follow-up or maintenance sessions to sustain benefits. As one professional proposed, *“Perhaps it would be good to have a session every month, not weekly. Some continuity would make it more meaningful”* (PC23, FG5).

They also highlighted the need for greater institutional support, particularly regarding protected time and appropriate spaces, noting that these conditions are essential for equitable access across centers.

RBECs highlighted the adaptability of the program to other settings: *“I think it could work very well elsewhere”* (RBEC3, FG1).

Finally, many participants stressed the importance of embedding emotional well-being practices into the everyday culture of primary care rather than offering them as isolated initiatives. As one participant summarized, “…these dynamics should always exist […]. If you don’t take care of yourself, you can’t care for others” (PC28, FG4).

## 4. Discussion

The findings of this study reveal how a psychoeducational program for emotional well-being in healthcare workers was received and implemented across diverse primary care settings. Overall, participants described the sessions as a valuable opportunity to pause, share emotions, and reconnect with strategies that support self-care, activities often displaced by the pressures of daily work. These insights reinforce the relevance of providing structured and protected spaces for emotional reflection and resilience-building within primary care teams, while also illustrating the challenges of embedding such initiatives in resource-constrained environments.

A central insight from participants’ accounts concerns the perceived role of managerial leadership in shaping implementation conditions. Participants described how center directors could facilitate participation by legitimizing the sessions, adjusting schedules, and ensuring adequate spaces, whereas inconsistent support was perceived as a barrier. This variability aligns with extensive research showing that emotionally supportive leadership fosters psychological safety, reduces burnout, and enhances team functioning, whereas indifferent or inconsistent leadership can undermine otherwise well-designed initiatives [21,22]. Although our participants tended to describe managerial support as a positive driver, they also noted that variations in commitment could meaningfully shape the program’s implementation and effects. These findings highlight that program outcomes depend not only on the content or on facilitation of the sessions but also on the organizational culture and leadership dynamics within each center. Given that managers were not interviewed, these findings should be interpreted as reflecting staff and RBEC perceptions of leadership-related factors rather than direct evidence of leadership effects.

Another recurring theme was the tension between strengthening individual resilience and addressing structural sources of distress. While participants valued practical self-care tools, some worried that such initiatives may shift responsibility for coping onto individuals when systemic contributors such as workload, staff shortages, administrative burden, and accumulated post-pandemic exhaustion remain insufficiently addressed. This concern aligns with the literature suggesting that individual-focused approaches are most effective when combined with organizational strategies targeting structural factors [13,23]. In line with this, our findings indicate that although the program was meaningful and valued, its longer-term impact may be limited without parallel organizational reforms. In this context, some accounts point to a risk of symbolic mitigation if such initiatives are institutionalized without those reforms. To situate this tension, we draw post hoc on the Job Demands–Resources model and related approaches, which emphasize that well-being reflects the interplay between workplace demands and available resources [24].

Despite these structural tensions, the program generated meaningful perceived effects at both interpersonal and team levels. Participants described the sessions as providing short-term relief and a protected space for reflection, and they perceived improvements in communication and cohesion within teams. These findings align with evidence that interdisciplinary emotional dialogue enhances collaboration and group cohesion, and that effective teams contribute to a more positive, engaging, and resilient workplace [25].

For RBEC psychologists, the program not only facilitated emotional expression among professionals but also strengthened their visibility and legitimacy within primary care teams, reinforcing their role as referents for emotional well-being. Their embedded position and continuity were perceived as supporting trust and acceptability, consistent with the literature highlighting the value of internal facilitators in implementation processes [21]. However, these perceived benefits may be time-limited and should not be interpreted as evidence of sustained change beyond the period of participation.

Challenges to implementation and sustainability, however, were substantial. Lack of protected time, competing workloads, and inadequate physical spaces constrained regular attendance and shaped unequal access across centers. These issues, operational in appearance, reflect the persistent fragility of primary care systems, where opportunities for reflection and team-based care are often secondary to immediate service demands. Some centers mitigated these constraints creatively by leveraging community resources, but such solutions further illustrate the uneven institutional support experienced. Similar international programs have reported early implementation fatigue or rapid discontinuation when logistical barriers outweighed initial enthusiasm, underscoring the importance of stable organizational commitment [21].

### 4.1. Contribution to Existing Knowledge

This study contributes to the literature in three ways. First, it provides empirical insight into how a large-scale emotional well-being program is implemented in real-world primary care, highlighting the mechanisms that enable or hinder adoption. Second, it demonstrates the value of embedded facilitators, such as RBEC psychologists, whose continuity and contextual knowledge appear critical for trust, engagement, and adaptation. Third, it clarifies the multi-level nature of implementation, showing how individual experiences, team dynamics, and organizational structures interact to shape feasibility and sustainability.

### 4.2. Limitations

Several limitations must be acknowledged. First, the perspectives represent a purposive, volunteer sample and may not reflect the views of all primary care professionals or psychologists; volunteer participation may have introduced self-selection, potentially under-representing more skeptical or disengaged views. Nevertheless, critical perspectives on structural constraints also emerged. Second, we did not assess profession-specific differences, and the number and distribution of participating professionals did not allow robust comparisons across professional groups. Third, the purposive sample may over-represent mid-career professionals, which may influence engagement with emotional well-being initiatives. Fourth, the use of online focus groups may have constrained spontaneity and limited access to non-verbal communication [26]. Fifth, managerial leaders or organizational decision-makers were not included, which constitutes a structural limitation for interpreting leadership-related influences on implementation; future research should incorporate these perspectives. Finally, transferability is likely greatest to health systems with similar organizational cultures and resource constraints.

### 4.3. Implications for Practice and Policy

The results suggest that emotional well-being initiatives should be embedded within broader organizational strategies rather than deployed as isolated activities. Consistent managerial endorsement, adequate physical spaces, and protected time within working hours appear to be key enabling conditions for sustainability and for scaling or transfer to other settings. These conditions may be easier to maintain when delivery is supported by embedded facilitators (e.g., RBEC psychologists) and complemented by periodic follow-up (“booster”) sessions. However, institutionalizing such programs without parallel organizational reforms addressing workload, staffing, and administrative pressures may be perceived as symbolic, may shift responsibility to individuals, and may exacerbate inequities in access across centers. Overall, a dual approach—supporting individuals while also modifying structural determinants—seems necessary to achieve sustained improvements in the emotional well-being of primary care professionals [14,23].

## 5. Conclusions

The psychoeducational emotional well-being program was well accepted by primary care professionals and offered a protected space for emotional expression, self-care, and stronger team cohesion. The embedded role of RBEC psychologists and the support of center leadership facilitated implementation, while workload pressures, limited protected time, and space constraints affected participation. Although participants noted short-term benefits, maintaining these effects over time will likely require continued organizational support. Overall, the findings underscore the value of integrating emotional well-being initiatives into routine practice and complementing individual-focused strategies with broader system-level support.

## Figures and Tables

**Table 1:** Characteristics of participants in the qualitative study.

	Community Psychologists (RBEC)	Primary Care Professionals
	N = 18	N = 20
Age (median, IQR)	38.5 (31–42.2)	44 (37.2–48.5)
Gender: female	16 (88.9%)	18 (90.0%)
Professional profile		
− Nurse	-―	11 (55.0%)
− Doctor	-―	4 (20.0%)
− Administrative staff	-―	4 (20.0%)
− Other	-―	1 (5.0%)
Territorial context		
− Urban	9 (50.0%)	12 (60.0%)
− Rural	9 (50.0%)	8 (40.0%)

Notes: Professional profile categories apply to the primary care professional group. In the Primary care professionals column, “Other” corresponds to a dental hygienist (n = 1).

**Table 2:** Content and structure of the psychoeducational program.

Topic	Description
Emotional management	Recognizing and regulating one’s own emotions.
Thought management	Inner dialogue, irrational beliefs, and the contrast between rational and distorted thoughts.
Stress management	Understanding stress, its causes, coping strategies, and its impact on health.
Communication skills	Assertiveness, communication styles, empathy, and active listening.
Self-care	Definition, importance, and the different dimensions of self-care.
Individual and group self-esteem	Concepts of self-esteem, its significance, relationship with self-concept, and traits of low self-esteem.
Anxiety and panic coping—mindfulness	Introduction to anxiety and stress concepts, along with basic mindfulness practices.
Motivation activation	Theoretical foundations of motivation and strategies for self-motivation.
Problem solving	Definition, conflict management, and effective problem-solving techniques.
Positive psychology and emotional intelligence	Positive emotions, resilience, positive work attitudes, engagement, and the flow experience.
Emotional expression through art	Health benefits derived from artistic expression.
Each session follows a consistent format, including: (1) an initial content presentation by the facilitator; (2) interactive activities to reinforce learning; (3) guided relaxation exercises; and (4) reminders about available participant resources.	

**Table 3:** Topic guide for the focus groups with primary care professionals and RBEC psychologists (summary).

Domain	Primary Care Professionals	RBEC Psychologists
1. Experience and overall appraisal	Experience participating in sessions; perceived usefulness of contents and group dynamics.	Experience delivering the program; appraisal of contents, tools, and institutional support.
2. Facilitators and barriers	Scheduling, workload, space, and compatibility with daily practice; examples of barriers/facilitators.	Organizational, logistical, and contextual barriers; facilitating elements within centers and teams.
3. Perceived usefulness and impact	Personal and professional usefulness; application to patient care and team functioning.	Acceptance among professionals; perceived needs addressed; aspects with greatest impact.
4. Integration of learning	Factors enabling or hindering integration into daily life; concrete examples.	Factors influencing effective delivery and team uptake; role of center dynamics.
5. Improvement proposals	Suggestions and prioritization of actions to enhance program usefulness and feasibility.	Proposed improvements to program design, support, and sustainability; unmet needs.
6. Additional topics	–	Value of the program as a “toolbox”; limitations regarding structural/organizational issues.

**Table 4:** Emergent themes from the qualitative analysis of the psychoeducational emotional well-being program.

Domain	Subthemes/Concepts	Analytical Description	Illustrative Quotes	Implementation Outcomes
1. Pre-existing conditions and emotional context	− High workload and post-pandemic emotional strain − Positive expectations toward the program − Uneven initial dissemination − Pre-existing team cohesion	Participants described pervasive emotional strain linked to workload and pandemic aftermath. The program was welcomed as an opportunity for self-care, although initial communication was sometimes insufficient. Cohesive teams tended to integrate the sessions more smoothly.	“COVID was tough enough already, because it also meant dealing with everything that suddenly hit all of us at once.” (PC28, FG4) “I felt there could have been more information; understanding our role was already difficult.” (RBEC7, FG1) “This time helped with group cohesion.” (PC33, FG3)	Adoption Acceptability
2. Facilitating factors and institutional support	− Active involvement of RBEC − Managerial support (schedules, space, visible endorsement) − Trust and familiarity with RBEC	RBEC psychologists were considered essential to engagement due to their familiarity with teams and facilitation skills. Managerial support—adjusting schedules and providing appropriate spaces—was decisive for participation. Trust, proximity, and continuity facilitated openness and motivation.	“The psychologist was very dynamic and approachable; that kept us motivated.” (PC25, FG5) “…the center’s management made it easy for us, during working hours, to avoid home visits or anything like that.” (PC24, FG5)	Feasibility Adoption Sustainability
3. Perceptions of content and group dynamics	− Preference for experiential activities − Materials perceived as too theoretical − Local adaptations − Value of multiprofessional groups	Participants valued practical exercises and experiential learning. Some sessions were initially perceived as too theoretical, prompting RBECs to adapt content. The multiprofessional format promoted mutual understanding, horizontal communication, and shared reflections.	“The presentations were very theoretical and long; I reduced the theory and focused on practice.” (RBEC4, FG1) “The most useful session was the last one, without slides.” (PC32, FG3) “…this relationship of mutual listening between each other, of mutual help, and of working together as a group.” (RBEC18, FG2)	Acceptability Feasibility
4. Perceived benefits and impact	− Space for emotional ventilation and relief − Learning/reinforcing self-care strategies − Improved team cohesion and mutual support − Recognition and visibility of RBEC role − Applicability in personal and professional life	The program provided emotional relief and reactivated self-care practices. Team cohesion improved as professionals shared experiences and concerns. RBECs gained visibility and legitimacy within teams. Participants highlighted the usefulness of tools beyond the sessions, in daily professional and personal contexts.	“Professionals realized that to care for others, they need to care for themselves.” (RBEC18, FG2) “We realized we needed a bit more cohesion.” (PC33, FG3) “Indirectly, I gained visibility in the primary care team.” (RBEC1, FG1) “It is a space that they also learn to apply to their day-to-day lives in times of need.” (RBEC9, FG1)	Acceptability Sustainability
5. Challenges and barriers	− Workload and lack of protected time − Inadequate physical spaces − Uneven institutional support − Overlap with other programs − Internal tensions and variable acceptance − Structural concerns beyond the scope of the program	Barriers mainly related to time constraints, workload, and space limitations. Uneven managerial engagement created substantial variation in implementation. Some questioned whether the program could compensate for broader structural issues, reflecting a tension between individual self-care and systemic determinants.	“Schedules weren’t closed, so even if they had the time, they couldn’t attend.” (RBEC5, FG1) “The space we used at the center lacked privacy due to a corridor and people passing by.” (RBEC13, FG2) “It was like putting a band-aid on a gunshot wound.” (PC30, FG4) “What we need more than this program is additional staff.” (RBEC15, FG2) “…they said things like, ‘I would rather you take care of the population than of us’.” (RBEC14, FG2)	Feasibility Adoption Sustainability
6. Improvement proposals and future perspectives	− Greater flexibility and local adaptation − Maintenance/booster sessions − Stronger institutional support (time, space) − Institutionalization as routine practice − Embedding emotional well-being in center culture − Potential expansion to other contexts	Participants proposed periodic follow-up sessions and flexible content tailored to team needs. They emphasized the importance of institutionalizing emotional well-being practices, ensuring protected time and adequate spaces, and integrating them into the organizational culture. The program was perceived as adaptable to other settings beyond primary care.	“Perhaps it would be good to have a session every month, not weekly. Some continuity would make it more meaningful” (PC23, FG5) “I think it could work well in other contexts.” (RBEC3, FG1) “…these dynamics should always exist […]. If you don’t take care of yourself, you can’t care for others.” (PC28, FG4)	Sustainability

Notes: RBEC is the acronym for ‘emotional well-being referent’, which is what the community psychologists who have taught the program are called. PC: primary care professional; numbers indicate anonymized participant codes. FG: focus group. Implementation outcome labels are used as a reporting map to link emergent themes to feasibility, acceptability, adoption, and sustainability; they did not guide data collection or coding.

## Data Availability

The data presented in this study are available on request from the corresponding author due to ethical and confidentiality restrictions. The datasets consist of verbatim transcripts from focus group sessions, which contain potentially identifiable and sensitive qualitative information. Public sharing of these data is not permitted under the terms of the informed consent approved by the ethics committee.

## Supplementary Materials

The following supporting information can be downloaded at: https://www.mdpi.com/article/10.3390/healthcare14030402/s1, COREQ Checklist: Consolidated Criteria for Reporting Qualitative Research.

## Abbreviations

The following abbreviations are used in this manuscript:

COREQ	Consolidated criteria for reporting qualitative research
COVID-19	Coronavirus disease 2019
FG	Focus group
ICS	Catalan Health Institute, in Catalan: Institut Català de la Salut
IDIAP	Primary Care Research Institute. In Catalan: Institut d’Investigació en Atenció Primària
RBEC	Community Emotional Well-being Lead. In Catalan: Referent de Benestar Emocional Comunitari
